# Mitotic spindle assembly and γ-tubulin localisation depend on the integral nuclear membrane protein Samp1

**DOI:** 10.1242/jcs.211664

**Published:** 2018-04-13

**Authors:** Veronica J. Larsson, Mohammed Hakim Jafferali, Balaje Vijayaraghavan, Ricardo A. Figueroa, Einar Hallberg

**Affiliations:** Department of Neurochemistry, Stockholm University, SE-106 91 Stockholm, Sweden

**Keywords:** Samp1, Nuclear membrane, Mitotic spindle, γ-Tubulin, Augmin, Cancer

## Abstract

We have investigated a possible role for the inner nuclear membrane protein Samp1 (also known as TMEM201) in the mitotic machinery. Live-cell imaging showed that Samp1a–YFP (Samp1a is the short isoform of Samp1) distributed as filamentous structures in the mitotic spindle, partially colocalising with β-tubulin. Samp1 depletion resulted in an increased frequency of cells with signs of chromosomal mis-segregation and prolonged metaphase, indicating problems with spindle assembly and/or chromosomal alignment. Consistent with this, mitotic spindles in Samp1-depleted cells contained significantly lower levels of β-tubulin and γ-tubulin, phenotypes that were rescued by overexpression of Samp1a–YFP. We found that Samp1 can bind directly to γ-tubulin and that Samp1 co-precipitated with γ-tubulin and the HAUS6 subunit of the Augmin complex in live cells. The levels of HAUS6, in the mitotic spindle also decreased after Samp1 depletion. We show that Samp1 is involved in the recruitment of HAUS6 and γ-tubulin to the mitotic spindle. Samp1 is the first inner nuclear membrane protein shown to have a function in mitotic spindle assembly.

## Abstract (precis)

**Highlighted Article:** The transmembrane inner nuclear membrane protein Samp1 has a functional role in recruitment of γ-tubulin to the mitotic spindle and correct spindle assembly.

## INTRODUCTION

In mammalian cells, a bipolar mitotic spindle is formed to segregate chromosomes between daughter nuclei of dividing cells. Failure to segregate chromosomes correctly will lead to aneuploidy, a signature of most cancers (Holland and Cleveland, 2012). The main elements of the complex spindle architecture are the spindle poles, the chromosomes and microtubules (Kline-Smith and Walczak, 2004; Meunier and Vernos, 2012). The nucleation of microtubules is initiated by γ-tubulin at the centrosomes, at the chromosomes and along the walls of existing microtubules (Goshima et al., 2008; Meunier and Vernos, 2012). The recruitment of γ-tubulin to different sites in the mitotic cell is regulated by different factors to ensure correct mitotic spindle assembly (Lin et al., 2015), although the exact mechanisms are not completely understood. The eight-subunit Augmin complex is involved in microtubule nucleation along the walls of existing microtubules (Goshima et al., 2008; Sánchez-Huertas and Lüders, 2015). Augmin has been shown to drive branching microtubule formation at low angles in the body of the spindle by recruiting γ-tubulin, and thus it rapidly increases the number of parallel microtubules in the spindle (Kamasaki et al., 2013; Petry et al., 2013; Zheng and Iglesias, 2013). Augmin binds to microtubules through its subunit HAUS8 (also known as Hice1) and recruits γ-tubulin via its subunit HAUS6 (also known as Dgt6), but how Augmin is recruited to spindle microtubules is not known (Hsia et al., 2014; Zhu et al., 2008). Disorganisation of the Augmin complex, HAUS6 and/or γ-tubulin results in reduced microtubule formation, as well as reduced microtubule bundling, affecting spindle assembly during mitosis and microtubule organisation during interphase (Goshima and Scholey, 2010; Sánchez-Huertas et al., 2016)

In open mitosis, the nucleus is disassembled in a process called nuclear envelope breakdown (NEBD) to give room for the mitotic spindle. Most transmembrane nuclear envelope proteins disperse out into the ER, the nuclear membrane loses its identity, and the nucleoplasm and cytoplasm blend (Ellenberg et al., 1997; Yang et al., 1997). The clearance of bulk membranes from the metaphase chromosomes has been shown to be important for correct chromosome segregation, and a model where ER membranes actively are transported from the metaphase plate towards each spindle pole through interactions with microtubules has been proposed (Schlaitz et al., 2013). However, membranes and a specific subset of transmembrane proteins, including Samp1 (also known as TMEM201, or Net5 in rat liver), TMEM214 and WFS1 have been shown to localise to the mitotic spindle (Buch et al., 2009; Lu et al., 2009; Wilkie et al., 2011; Schirmer et al., 2003). A functional role for these spindle-associated transmembrane proteins has not yet been defined. The transmembrane protein Samp1 (Buch et al., 2009; Gudise et al., 2011) was identified in two genome-wide siRNA screens for proteins involved in mitosis in *C. elegans* (Sonnichsen et al., 2005) and HeLa cells (Neumann et al., 2010). Here, we have tested a potential role for the short isoform of Samp1, Samp1a (Borrego-Pinto et al., 2012; Buch et al., 2009), in the mitotic machinery.

## RESULTS

### The transmembrane protein Samp1 is present as filamentous structures along microtubules of the mitotic spindle

**Fig. 1.**
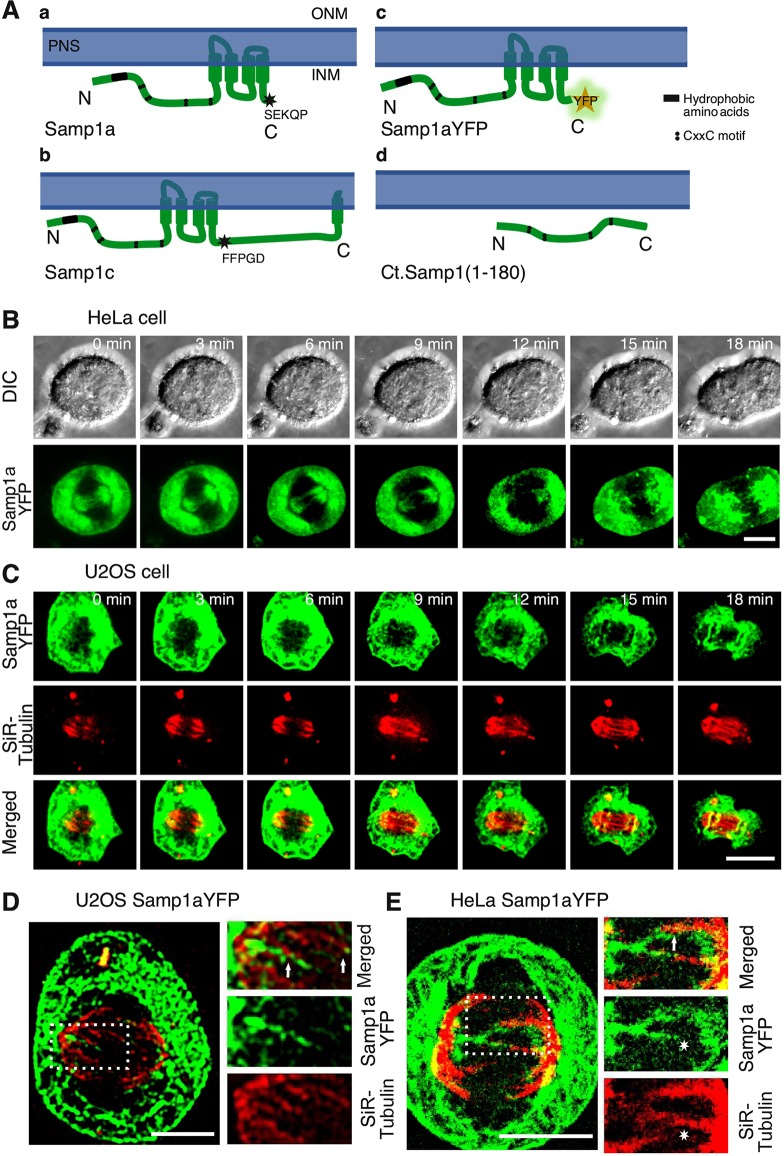
**Live-cell imaging of Samp1a–YFP distribution in the mitotic spindle.** (A) Schematic illustration of validated isoforms Samp1a (a) and Samp1c (b), which have identical N-terminal domains with a hydrophobic region (black box) and four conserved CxxC motifs (black circles). The shorter Samp1a (392 amino acids, NM_001010866.3) has a short C-terminal. The longer Samp1c (666 aa, NM_001130924.2) has a long C-terminal tail and one extra transmembrane segment close to the C-terminus. Five amino acids differ between the two isoforms, indicated by the black stars. (c) Samp1a was recombinantly tagged with yellow fluorescent protein (YFP). (d) The soluble N-terminal domain of the Samp1 homologue in *Chaetomium thermophilum* (*Ct.*), which was used in *in vitro* pulldown assays using recombinant proteins. (B) Time-lapse images of a mitotic HeLa cell stably expressing Samp1a–YFP. Confocal laser scanning microscopy fluorescence and phase-contrast stills are shown. Time is indicated in the upper left corner. See also Movie 1. Scale bar: 10 µm. (C) Time-lapse images of a mitotic U2OS cell stably expressing Samp1a–YFP (green) and probed with SiR–tubulin to visualise microtubules (red). See also Movie 2. Scale bar: 10 µm. (D) The image taken at 9 min from the time-lapse presented in B was enhanced by performing convolution followed by a Gaussian blur filter to better visualise the parallel elongated structures of microtubules (red) and Samp1a–YFP (green). Scale bar: 5 µm. The inset shows an enlargement, and arrows show overlap between the Samp1 and microtubule stains. (E) De-convolved image stack of a live mitotic HeLa cell. Samp1a–YFP (green) distributes in filamentous structures in parallel with microtubules (arrow) and in apparent contact with microtubules (asterisk). See also Movie 3. Scale bar: 5 µm.

There are two validated isoforms of Samp1, the short Samp1a and the longer Samp1c (Fig. 1Aa,b). The nucleoplasmically exposed N-terminal domain shared by both splice variants, contains a hydrophobic segment and four conserved CxxC motifs (Buch et al., 2009; Gudise et al., 2011). Samp1a has four transmembrane segments whereas Samp1c has five transmembrane segments. Here, we used human HeLa and U2OS cell lines stably expressing Samp1a–YFP (Fig. 1Ac). The recombinant protein expression levels were ∼4 times higher than endogenous Samp1 expression levels (Fig. S1). In order to document the distribution and dynamic behaviour of Samp1 in live mitotic cells, we recorded time-lapse movies. HeLa cells stably expressing Samp1a–YFP (Fig. 1Ac) were synchronised at the G2/M boundary by treatment with the CDK1 inhibitor RO-3306 overnight, and released for 2–3 h before imaging. Images from a time-lapse series are shown in Fig. 1B and Movie 1. During metaphase and anaphase, Samp1a–YFP was most abundant in the ER, but a substantial fraction had a poleward localisation in the mitotic spindle, whereas a smaller fraction localised as elongated filamentous structures apparently spanning from spindle pole to spindle pole (Fig. 1B). In telophase, Samp1a–YFP was recruited to the re-forming nuclear envelope. To visualise Samp1a–YFP distribution compared to microtubules of the mitotic spindle, we probed for microtubules by using the *in vivo* dye SiR–tubulin. Images from a time-lapse series of a mitotic U2OS cell shows that Samp1a–YFP (green) was present as filamentous structures parallel to microtubules (red) (Fig. 1C; Movie 2). Images from the time-lapse series were analysed in greater detail using the software ImageJ to remove background noise and enhance the structures revealed by Samp1a–YFP and SiR–tubulin. Image convolution followed by a Gaussian Blur filter (Fig. 1D) shows that Samp1a–YFP and microtubules were present as parallel filamentous structures (arrows). Image de-convolution of a metaphase HeLa cell (Fig. 1E; Movie 3) shows that Samp1a–YFP localised parallel to microtubules and spanned almost the entire length of the spindle. To summarise, live cell imaging of two different cell types shows that the transmembrane protein Samp1a–YFP is present in elongated filamentous structures in the mitotic spindle that are parallel to and occasionally colocalize with microtubules. This is consistent with the localisation of endogenous Samp1 during mitosis (Buch et al., 2009). This prompted us to elucidate what function Samp1 has in the mitotic spindle.

### Depletion of Samp1 results in aneuploidy phenotypes

**Fig. 2.**
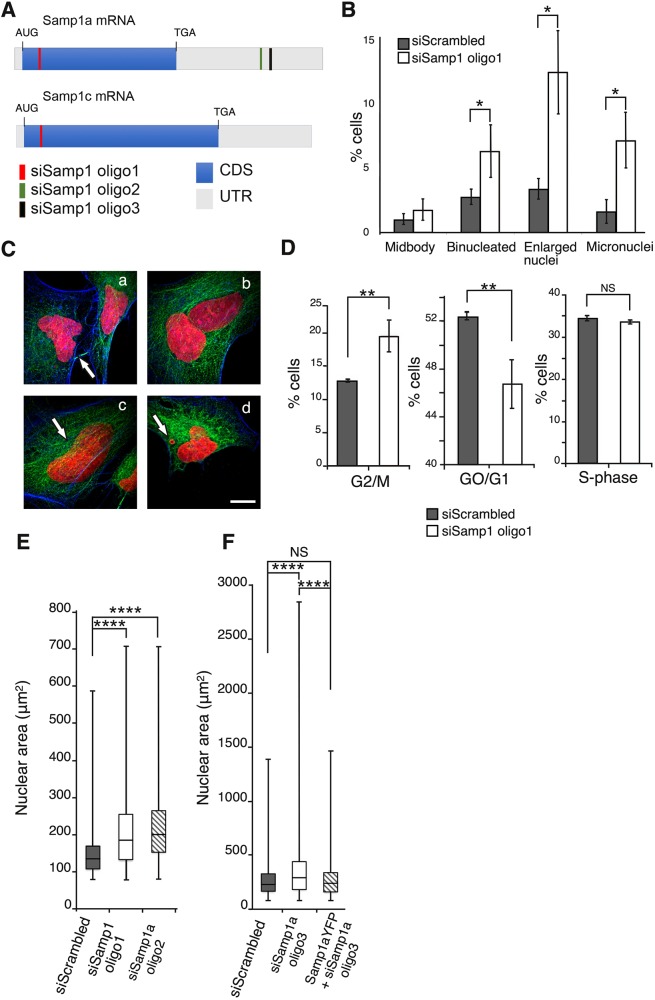
**Nuclear phenotypes in Samp1-depleted cells.** (A) Schematic illustration of Samp1 mRNA isoform 2 (Samp1a, NM_001010866.3) and isoform 1 (Samp1c, NM_001130924.2). The UTR (grey), coding DNA sequence (CDS, blue) and the positions of Samp1-specific siRNA oligonucleotides are indicated. HeLa cells were treated with siRNA against Samp1 (siSamp1) or scrambled control siRNA (siScrambled) and stained for DNA (Hoechst 33342, red) and actin (phalloidin, blue), and immunostained using antibodies specific for tubulin (green). (B) The graph shows the frequency of observed phenotypes expressed as the percentage from triplicate experiments (*n*=500 cells; mean±s.d.; *P<0.05, unpaired two-tailed Student's *t*-test). The Samp1-depleted cell population (siSamp1) displayed a significant increase in the occurrence of bi-nucleated cells, cells with enlarged nuclei and cells with micronuclei. (C) The cells were classified into the different categories as highlighted by the arrows (a, midbody; b, bi-nucleated; c, enlarged nuclei; d, micronuclei). Scale bar: 20 µm. (D) Graphs showing compilation of FACS data from triplicate experiments (*n*=10,000 cells; mean±s.d.; **P<0.01, NS, not significant, unpaired two-tailed Student's *t*-test), of control (siScrambled) and Samp1-depleted (siSamp1) cells. Samp1 depletion resulted in a significant increase of cells in G2/M, a significant decrease of cells in G0/G1, and no significant difference of cells in S-phase. (E) Nuclear area distribution from siScrambled control cells (*n*=3045 cells) and Samp1-depleted cells (siSamp1 oligo 1 and siSamp1 oligo 2, *n*=3045 cells). Samp1-depleted cells displayed a significantly increased fraction of abnormally large nuclei (****P<0.0001, unpaired two-tailed Student's *t*-test). (F) The distribution of the nuclear area in the siScrambled (blue, *n*=1940), siSamp1a oligo 3 (*n*=2400) and in Samp1a–YFP+siSamp1a oligo 3 (*n*=3100) cell population, respectively. Samp1-depleted cells displayed a significantly increased fraction of abnormally large nuclei compared to siScrambled (****P<0.0001) and was rescued by Samp1a–YFP (****P<0.0001, unpaired two-tailed Student's *t*-test). Box plots are presented as described in the Materials and Methods.

In order to investigate whether Samp1 has a role in the mitotic machinery, we treated cells with siRNA (Fig. 2A) for 96 h necessary to silence its expression (Buch et al., 2009; Gudise et al., 2011) and looked for phenotypes. After 96 h, Samp1-depleted HeLa cells displayed a significant 2-fold increase in the frequency of bi-nucleated cells (Fig. 2B,Cb), a 3-fold increase in the frequency of cells with enlarged nuclei (Fig. 2B,Cc) and in cells with micronuclei (Fig. 2B,Cd). These phenotypes are indicative of incorrect chromosome segregation and/or failed cytokinesis (Holland and Cleveland, 2012; Shi and King, 2005). The slight increase in the percentage of cells with a persistent midbody after Samp1 depletion (Fig. 2B,Ca), was not statistically significant. FACS analysis of the DNA content from siRNA-treated cell populations revealed that the Samp1-depleted cell population contained a significantly higher percentage of cells with a higher DNA content, and were classified as being in G2/M by the analysis software (Fig. 2D). The percentage of cells in G2/M increased from 12.5% to 20% in the Samp1-depleted cell population whereas the percentage of cells in G0/G1 decreased from 52% in the control cell population to 46% in Samp1-depleted cells (Fig. 2D). The percentage of cells in S-phase did not significantly differ between the two groups (Fig. 2D). The apparent accumulation in G2/M and the loss of cells in G0/G1 might be explained both by an increase in binucleated (from 2.7% to 6.3%, Fig. 2Cb) cells and nuclei with abnormally high DNA content (from 3.4% to 12.5%, Fig. 2Cc), rather than a block in cell cycle progression. In order to achieve an unbiased measure of the frequency of abnormal nuclei, we performed automated image sampling and programed determination of nuclear areas by using the image analysis software CellProfiler (Carpenter et al., 2006; Kamentsky et al., 2011). The results show that HeLa cells post-transcriptionally silenced for either the short Samp1a isoform (oligo 2 or 3) or all Samp1 isoforms (oligo 1) had a higher frequency of nuclei with abnormally high DNA content (Fig. 2E,F). This phenotype was completely rescued by simultaneous overexpression of siRNA resistant Samp1a–YFP (Fig. 2F). Rescue was efficient using the short isoform Samp1a, even in cells depleted of both isoforms. Overall our results are consistent with the appearance of cells with higher DNA content (binucleated cells, cells with enlarged nuclei and cells with micronuclei) seen in Fig. 2B, suggesting potential defects in chromosome segregation and/or cytokinesis.

### Depletion of Samp1 leads to metaphase prolongation and defective mitosis

**Fig. 3.**
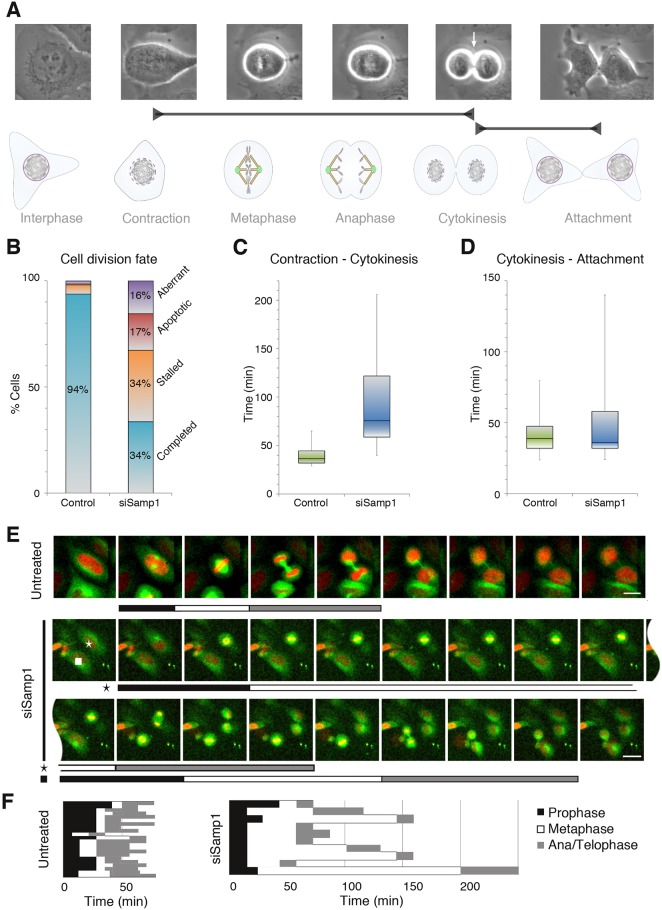
**Samp1 is required for normal progression of cell division and Samp1-depleted cells display a specific metaphase prolongation.** Dividing HeLa cells were monitored by phase-contrast time-lapse microscopy. (A) Phase-contrast images of typical stages of cell division and schematic illustration. The arrow indicates cleavage furrow ingression (start of cytokinesis). (B) Graph showing the fate of dividing cells treated with scrambled (left, *n*=200 cells) or Samp1-specific siRNA (siSamp1; right, *n*=200 cells). Normal cell division, blue; cells with rounded up morphology for more than 60 min (stalled), orange; apoptotic cells, red; aberrant cell divisions, purple. Abnormal cell divisions increased significantly (*P*<0.001, unpaired two-tailed Student's *t*-test) in cells treated with siSamp1. (C) Graph showing the time duration from cell contraction to start of cytokinesis in cells treated with scrambled (green, *n*=200 cells) or Samp1-specific siRNA (blue, *n*=200 cells). Depletion of Samp1 resulted in a substantial prolongation of mitosis (*P*<0.001, unpaired two-tailed Student's *t*-test). (D) Graph showing the time duration from start of cytokinesis to attachment of daughter cells in cells treated with scrambled (green, *n*=200 cells) or Samp1-specific siRNA (blue, *n*=200 cells). The distribution (whiskers), median (horizontal bar) and the 25–75th percentile (box) are indicated. (E) HeLa cells stably expressing H2B–mCherry and tubulin–GFP were treated with Samp1-specific siRNA. Images from two representative time-lapse experiments showing dividing cells from control and siSamp1 treatment. Black, white and grey bars indicate prophase (black), metaphase (white) and ana/telophase (grey). The star and square denote the cell cycle phases of the two indicated Samp1-depleted cells, respectively, from the same time-lapse series. Scale bars: 15 µm (control); 30 μm (siSamp1). (F) A summary diagram of the duration of mitotic phases from different experiments shows that depletion of Samp1 results in a significantly prolonged metaphase, whereas duration of prophase and ana/telophase are unaffected.

In order to get a hint of what role Samp1 might have in mitosis, all isoforms of Samp1 were post-transcriptionally silenced by siRNA in HeLa cells. We then used phase-contrast time-lapse microscopy to monitor mitosis and cytokinesis of monolayer cultures of HeLa cells treated with scrambled control siRNA or siRNA specific for Samp1 (Fig. 3). The mitotic cells were treated with siRNA for 90 h prior to visualisation and monitored for at least 6 h. We defined the onset of mitosis by a rounded up cell morphology (contraction), the onset of cytokinesis as ingression of cleavage furrow, the end of cytokinesis as appearance of two separated cells, and the end of cell division as the attachment of two daughter cells with flattened morphology (Fig. 3A). Only 34% of Samp1-depleted cells completed cell division as compared to 94% among control cells (Fig. 3B, *n*=200 cells in each condition). Samp1 depletion resulted in a significant (*P*<0.001) 10-fold increase in the percentage of cells with abnormal division: 34% were stalled in mitosis or cytokinesis, 17% were apoptotic, and 16% had other aberrant phenotypes, most often cleavage furrow reversion and asymmetric cell attachment (only one daughter cell with flattened morphology) (Fig. 3B). Among the subset (34%) of Samp1-depleted cells that completed cell division, we detected a significant (*P*<0.001) 2-fold increase in the duration of contraction to cytokinesis (Fig. 3C), with no significant change in the duration of cytokinesis to attachment (Fig. 3D). Taken together, the results provide evidence that Samp1 has an important function in mitosis. To study the effect of Samp1 depletion in greater detail, we employed HeLa cells stably expressing GFP–tubulin and H2B–mCherry to enable simultaneous visualisation of the microtubule cytoskeleton and chromosomes by using time-lapse confocal laser scanning microscopy. We monitored cells that were going through mitosis (Fig. 3E) and measured the duration of prophase, metaphase, and anaphase and telophase together (ana/telophase). Depletion of Samp1 resulted in a significant (*P*<0.005) 6-fold prolongation of the metaphase, from a mean time of 12 min in controls to 74 min (Fig. 3F) in Samp1-depleted cells. As no significant differences in the duration of prophase or ana/telophase were observed, we conclude that Samp1 depletion specifically affects the metaphase-anaphase transition, implying potential defects in kinetochore capture or mitotic spindle assembly or stability (Foley and Kapoor, 2012; Prosser and Pelletier, 2017).

### Spindle assembly is negatively affected in Samp1-depleted cells

**Fig. 4.**
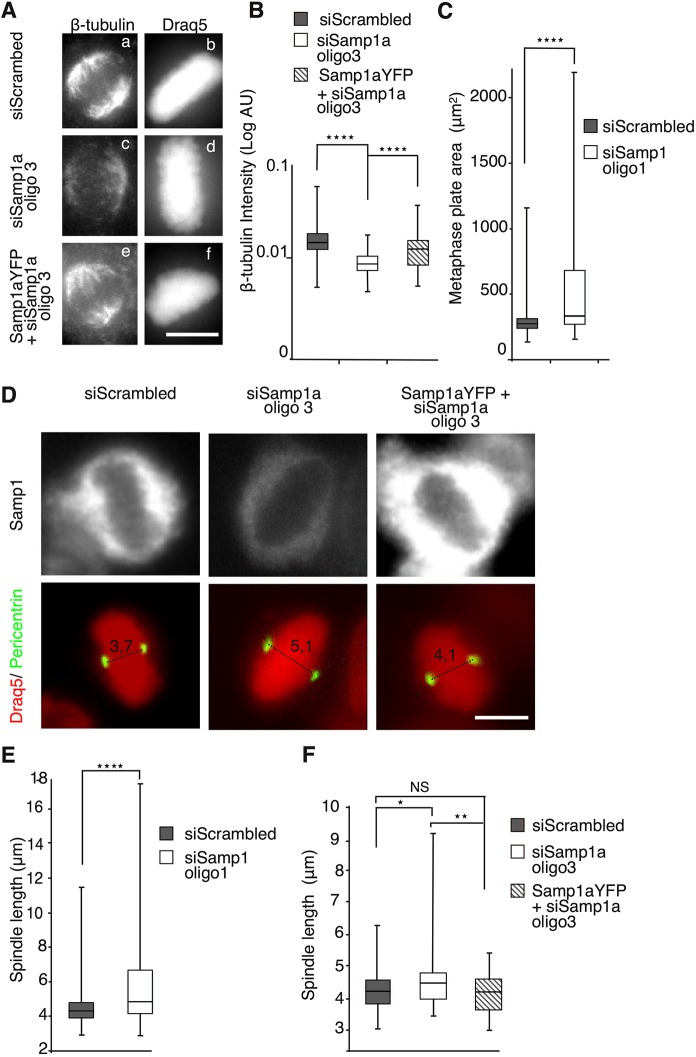
**Deficient mitotic spindle assembly in Samp1-depleted cells.** HeLa cells treated with siRNA and synchronised in metaphase were immunostained using antibodies specific for β-tubulin and pericentrin, and DNA was stained with DRAQ5. (A) Representative images of β-tubulin staining of metaphase spindles in cells treated with control, scrambled siRNA (siScrambled) or siRNA against Samp1 (siSamp1). Scale bar: 10 µm. (B) Quantification of β-tubulin immunofluorescence intensities from siScrambled (grey), siSamp1a oligo3 (white) and Samp1a–YFP+siSamp1a oligo3 (striped) metaphase spindles (*n*=150 cells, ****P<0.0001, unpaired two-tailed Student's *t*-test). AU, arbitrary units. (C) Quantification of the metaphase plate area, measured as *z*-stack maximum projections of HeLa cells treated with siScrambled (grey) or siSamp1 oligo1 (white) (****P<0.0001, *n*=50 cells, four replicates, unpaired two-tailed Student's *t*-test). (D) Spindle length. Representative images showing pericentrin and Samp1 immunostaining in siRNA-treated cells in metaphase. The spindle pole-to-pole distances were measured. Scale bar: 10 µm. (E) Quantification of spindle pole-to-pole distance as shown in D, which was significantly increased in Samp1-depleted cells (white) compared to control cells (grey) (****P<0.0001, *n*=50 cells, three replicates, unpaired two-tailed Student's *t*-test). (F) Quantification of metaphase spindle pole-to-pole distance in cells treated with yet another siRNA oligonucleotide pair specific for Samp1a showed a significant increase in spindle length between siSamp1a-treated cells (white) compared to siScrambled (grey). Co-transfection of HeLa cells with cDNA encoding siRNA resistant Samp1a–YFP and siSamp1a (striped) rescued the increased spindle length phenotype (*P<0.05, **P<0.01; NS, not significant; *n*=150 cells, three replicates, unpaired two-tailed Student's *t*-test). Box plots are presented as described in the Materials and Methods.

To investigate potential structural defects in the mitotic spindle, HeLa cells were treated (96 h) with siRNA against Samp1a and synchronised in metaphase by first blocking the cells at the G2/M boundary by using the CDK1 inhibitor RO-3306 followed by release in medium containing the proteasome inhibitor MG132. The cells were then fixed and stained to visualise microtubules (anti-β-tubulin) and the metaphase plate (DNA stain DRAQ5) (Fig. 4A), or the centrosomes (anti-pericentrin) and endogenous Samp1 (Fig. 4D). Regardless of treatment, all cells had apparently normal bi-polar spindles (Fig. 4Aa–f), although Samp1a-depleted cells displayed a lower intensity of β-tubulin immunofluorescence (Fig. 4Ac,d). The β-tubulin intensity in Samp1a-depleted cells was significantly decreased, an effect that was rescued by ectopic expression of siRNA resistant Samp1a–YFP (Fig. 4Ae,f,B). Considering the knockdown of Samp1 using siRNA oligonucleotides is incomplete and the fact that all cells (even non-transfected cells) were counted, this is a substantial effect. The decreased β-tubulin intensity level is a clear indication of defective spindle assembly. In accordance with this result, the metaphase plate area significantly increased after Samp1 (oligo1) depletion (Fig. 4C); however, in principle this could also result from altered DNA condensation. We also measured the spindle pole-to-pole distance (spindle length) after Samp1 (oligo 1) and/or Samp1a (oligo 3) depletion. Representative images from Samp1 depletion (Fig. 4D, oligo3) and quantification (Fig. 4E, oligo1, F, oligo3) show that the spindle length significantly increased in Samp1-depleted cells compared to that found in control cells. The increased spindle lengths after Samp1a depletion was completely rescued by Samp1a–YFP overexpression (Fig. 4F). Taken together, the results show that Samp1 is required, and that the Samp1a isoform is sufficient for correct assembly of the mitotic spindle.

### Samp1 interacts directly with γ-tubulin necessary for microtubule nucleation

**Fig. 5.**
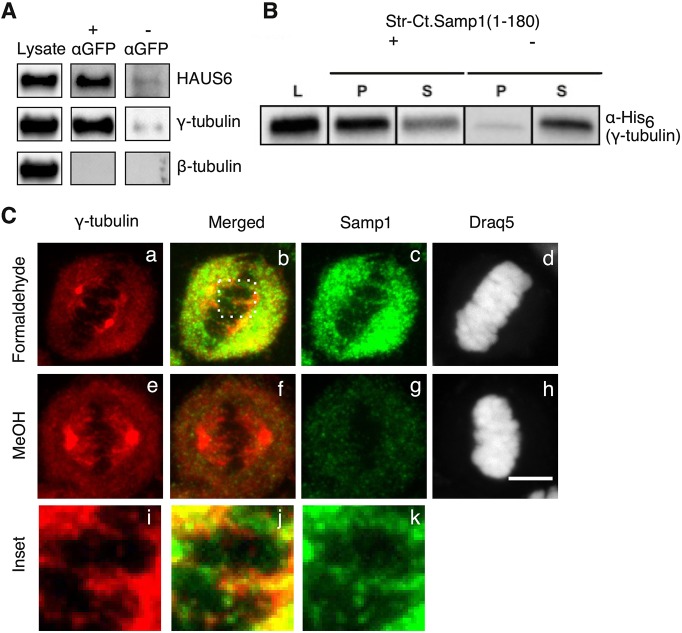
**Samp1 interacts directly with γ-tubulin.** HeLa cells stably expressing Samp1a–YFP were synchronised in metaphase. (A) MCLIP identified γ-tubulin and the HAUS6 subunit of the Augmin complex as interacting partners of Samp1a. (B) Equimolar amounts of recombinantly expressed Strep-*Ct*.Samp1(1-180) and His_6_–γ-tubulin were subjected to pulldown experiments using strep-tactin beads. Equivalent amounts of lysate (L), bound (P) and unbound (S) fractions were separated using SDS-PAGE and analysed by western blotting using anti-His_6_ antibody as indicated. (C) Metaphase HeLa cells were fixed using either 4% formaldehyde (a–d) or MeOH (e–h) and immunostained with antibodies specific for γ-tubulin and Samp1. DNA was stained with DRAQ5. The images shown in i–k are enlargemnents of the square area indicated by white dots in b showing that Samp1 and γ-tubulin partially colocalise. Scale bar: 10 µm.

To understand how Samp1 can contribute to mitotic spindle assembly and/or stability we used membrane protein cross-link immunoprecipitation (MCLIP; Jafferali et al., 2014) to test potential interactions between Samp1 and candidate proteins involved in spindle assembly. HeLa cells stably expressing Samp1a–YFP were synchronised in metaphase and subjected to MCLIP and analysed by performing SDS-PAGE and western blotting. Samp1a–YFP did not interact with β-tubulin, but instead bound γ-tubulin (Fig. 5A). Since, γ-tubulin is known to interact with the HAUS6 subunit of the Augmin complex we also tested whether HAUS6 co-precipitated with Samp1, which indeed it did (Fig. 5A). Thus, Samp1 interacts with two components that stimulate nucleation of microtubules on pre-existing microtubules in the body of the spindle (Goshima et al., 2008; Petry et al., 2013). The interaction with γ-tubulin was investigated in more detail by means of *in vitro* pulldown experiments using recombinantly expressed Samp1 and γ-tubulin. Since we were unable to express human Samp1 in soluble form, we used the nucleoplasmic N-terminal domain (amino acids 1–180) of the Samp1 homologue from *Chaetomium thermophilum*, here called Ct.Samp1(1-180) (Fig. 1Ad), which contains the evolutionarily conserved CxxC motifs (Buch et al., 2009; Vijayaraghavan et al., 2016). The results show that Samp1 binds γ-tubulin directly (Fig. 5B). We used immunofluorescence confocal microscopy to compare the localisation of Samp1 and γ-tubulin in mitotic HeLa cells (Fig. 5C). Methanol fixation is excellent for visualising γ-tubulin in spindles (Fig. 5Ce), but inadequate for visualising the integral membrane protein Samp1 (Fig. 5Cg), likely due to poor membrane preservation (Hoetelmans et al., 2001; Hua and Ferland, 2017). The merged image from a formaldehyde fixed mitotic HeLa cell (Fig. 5Cb) shows that Samp1 and γ-tubulin distributed in a similar pattern to each other in the body of the spindle. The structures in the boxed region (Fig. 5Cb) are enlarged in Fig. 5Ci–k and show overlapping distribution of Samp1 and γ-tubulin. From these data, we conclude that Samp1 interacts directly with γ-tubulin, and that Samp1 and γ-tubulin have overlapping localisations in the mitotic spindle. Direct binding between Samp1 and HAUS6 was not tested.

### Failed recruitment of γ-tubulin in the mitotic spindle after Samp1 depletion

**Fig. 6.**
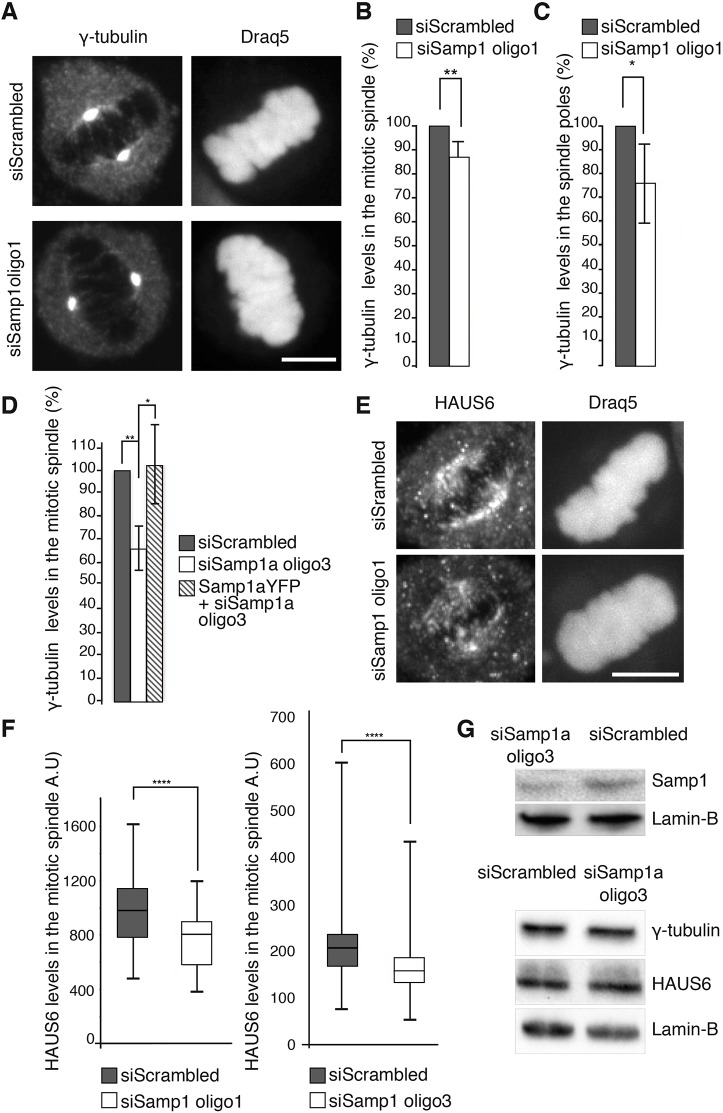
**The levels of γ-tubulin and HAUS6 in the mitotic spindle decrease after Samp1a depletion.** Synchronised HeLa cells were subjected to treatement with control, scrambled siRNA (siScrambled) or siRNA against Samp1 (siSamp1) and immunostained with antibodies specific for γ-tubulin and HAUS6. (A) Representative images of the distribution of γ-tubulin after siSamp1 and siScrambled siRNA treatment, as indicated. Scale bar: 10 µm. (B) Quantification of γ-tubulin fluorescence intensity in the mitotic spindle (**P<0.01, *n*=50 cells, three replicates, mean±s.d., unpaired two-tailed Student's *t*-test). (C) Quantification of γ-tubulin fluorescence intensity in the centrosomes (*P<0.05, *n*=50 cells, three replicates, mean±s.d., unpaired two-tailed Student's *t*-test). (D) The decreased levels of total (centrosome+spindle) γ-tubulin in the mitotic spindle after Samp1a depletion were rescued by Samp1a–YFP overexpression (*P<0.05, **P<0.01, *n*=20 cells, three replicates, mean±s.d., unpaired two-tailed Student's *t*-test). (E) Representative images of the distribution of Augmin (HAUS6) after siRNA treatment in the mitotic spindle. Scale bar: 10 µm. (F) Quantification of Augmin (HAUS6) fluorescence intensity in the mitotic spindle after siRNA treatment (oligo1, ****P<0.0006, *n*=26 cells, two replicates; oligo3, ****P<0.0005, *n*=53 cells, unpaired two-tailed Student's *t*-test). Box plots are presented as described in the Materials and Methods. A.U., arbitrary units. (G) Protein levels in mitotic cells treated with siRNA were analysed by SDS-PAGE and western blotting. The blots (representative of *n*=3 blots) were probed with antibodies specific for γ-tubulin, HAUS6 and Samp1. Lamin-B1 was used as loading control.

Because γ-tubulin, which is important for correct spindle assembly, interacted with Samp1a during mitosis, we wanted to investigate their functional connection in greater detail. Metaphase HeLa cells were immunostained with antibodies specific for γ-tubulin (Fig. 6A). The immunofluorescence intensity from γ-tubulin in the mitotic spindle (Fig. 6B) and spindle poles (Fig. 6C) significantly decreased after Samp1 depletion, an effect that was completely rescued by overexpression of siRNA-resistant Samp1a–YFP (Fig. 6D). Samp1a-depleted cells also displayed a significant decrease in HAUS6 immunofluorescence intensity in the mitotic spindles with two independent siRNA oligonucleotides for Samp1 (Fig. 6E,F). The total protein levels of γ-tubulin and HAUS6 in siRNA-treated mitotic cells were unaffected as shown by SDS-PAGE followed by western blotting (Fig. 6G). We therefore conclude that Samp1 depletion affects the distribution and not the levels of γ-tubulin and HAUS6 in the mitotic spindle. Microtubule minus-ends are sorted and transported poleward from the body of the spindle, leading to the accumulation of γ-tubulin at the centrosomes (Lecland and Lüders, 2014). We speculate that the decreased levels of γ-tubulin in the centrosome after Samp1 depletion (Fig. 6A,C), is most likely a consequence of failed recruitment of γ-tubulin into the body of the spindle.

## DISCUSSION

The recruitment of transmembrane proteins to filamentous structures in the mitotic spindle has previously been demonstrated (Buch et al., 2009; Lu et al., 2009; Wilkie et al., 2011), but a functional role for these proteins in mitosis has not yet been defined. Here, we present data showing that a subpopulation of the inner nuclear membrane protein Samp1, distributes as filamentous structures in parallel with microtubules of the mitotic spindle in live HeLa and U2OS cells during metaphase (Fig. 1). Samp1 depletion prolonged metaphase and elongated the mitotic spindle, resulting in a less compact and less well-organised metaphase plate (Fig. 4) and an increased percentage of cells with signs of chromosome mis-segregation (Fig. 2). All these phenotypes are signs of reduced mitotic spindle stability. In support of this conclusion, we observed a reduction in β-tubulin fluorescence intensity in the mitotic spindle after Samp1 depletion (Fig. 4), which strongly indicates that the number of microtubules has been reduced (Goshima and Scholey, 2010). Interestingly, our experiments show that, during mitosis, Samp1 binds directly to γ-tubulin (Fig. 5), which is necessary for microtubule nucleation. The interaction with γ-tubulin and HAUS6 of the Augmin complex and these characteristic phenotypes indicate that Samp1 contributes to the assembly and/or stability of the mitotic spindle.

The mitotic phenotypes seen when Samp1 was downregulated (Figs 4 and 6) were moderate (not lethal), consistent with incomplete knockdown and the fact that all cells (even non-transfected cells) were counted. Mild disturbances of the mitotic process are relevant for tumour development. The delayed metaphase-to-anaphase transition (Fig. 3E,F), indicates that the spindle assembly checkpoint is not satisfied. Since this checkpoint is relatively weak, many cells eventually proceed into anaphase (Rieder and Maiato, 2004), consistent with the phenotypes observed in Samp1-depleted cells. We therefore speculate that Samp1 defects, over time, might lead to chromosome instability and contribute to cancer (Holland and Cleveland, 2012; Silk et al., 2013).

The recruitment of γ-tubulin by Augmin in the spindle is important for assembling normal length bipolar spindles with properly aligned chromosomes (Goshima et al., 2008; Uehara et al., 2009). Depletion of Augmin has been shown to cause reduced microtubule density in the spindle, metaphase prolongation, chromosome mis-segregation, mis-localisation of γ-tubulin, elongated spindles and cytokinesis failure (Goshima et al., 2008, 2007; Uehara et al., 2009). These phenotypes are also seen after Samp1 depletion, supporting our model that Samp1 has a functional role in recruitment of γ-tubulin to the mitotic spindle. The decreased recruitment of γ-tubulin and Augmin in the spindles of Samp1-depleted mitotic cells (Fig. 6) can explain the spindle defects and chromosome mis-segregation we observed. Augmin-dependent microtubule nucleation is promoted by Ran-GTP and TPX2 (Petry et al., 2013). Although, we have shown that Samp1 can bind to Ran (Jafferali et al., 2014; Vijayaraghavan et al., 2016), Ran localisation in mitotic spindles appeared normal after Samp1 depletion (data not shown).

Potential roles of membranes in the mitotic spindle have been discussed by others (Meunier and Vernos, 2012; Rebollo et al., 2004; Tsai et al., 2006; Zheng, 2010), and our results provide new insight into the roles of membranes in the mitotic spindle. Previously, γ-tubulin has been shown to associate with membranous structures of the nuclear envelope both during interphase and mitosis, and Augmin is involved in the recruitment of γ-tubulin to membranes of the plant cell cortex (Binarová et al., 2000; Lesca et al., 2005; Liu et al., 2014; Macurek et al., 2008; Rosselló et al., 2016). Membrane-associated γ-tubulin complexes are able to initiate the formation of microtubules (Macurek et al., 2008). Furthermore, microtubule nucleation can occur near fenestrated membrane remnants from the NEBD, and these microtubules can be assembled into bipolar spindles (Rebollo et al., 2004). Our results support the hypothesis of membrane-dependent microtubule nucleation in human cells.

**Fig. 7.**
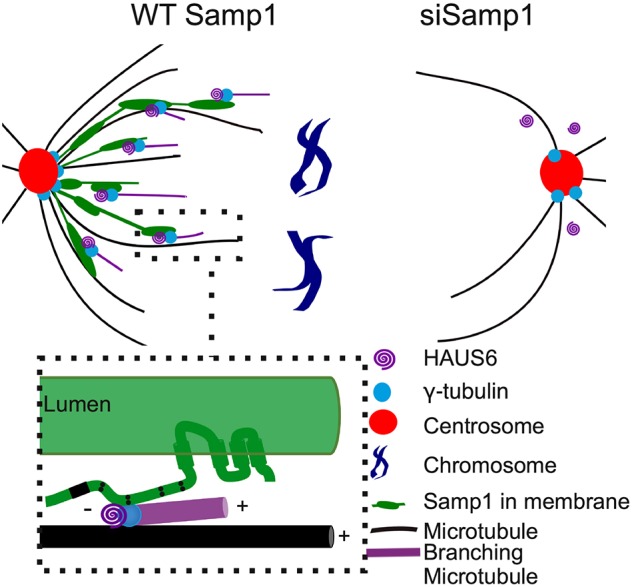
**Hypothetic model of the function of Samp1 in the mitotic spindle.** Samp1 is present as filamentous structures that are parallel to spindle microtubules and, recruits HAUS6 and γ-tubulin for microtubule nucleation during spindle assembly (inset). In Samp1-depleted mitotic cells, decreased recruitment of HAUS6 and γ-tubulin to the spindle results in fewer microtubules.

After NEBD, a fraction of Samp1 distributes as filamentous structures in the mitotic spindle. We hypothesise that Samp1 recruits γ-tubulin, HAUS6 and the Augmin complex, and thus increases the local concentrations of γ-tubulin along filamentous membranes in the mitotic spindle (Fig. 7, left). In Samp1-depleted cells (Fig. 7, right), less γ-tubulin is recruited to the mitotic spindle, resulting in fewer microtubules.

Microtubules of the mitotic spindle can be nucleated via centrosomal-, chromosomal- or Augmin-dependent pathways. All pathways recruit the microtubule nucleation factor, γ-tubulin. Here, we present evidence for the involvement of an additional component, the transmembrane inner nuclear membrane protein Samp1, to assembling spindles needed for efficient chromosome segregation. Future investigations into how membranes and transmembrane proteins are involved in spindle assembly are required to elucidate this mechanism in greater detail.

## MATERIALS AND METHODS

### Cell cultures

In this study two cell lines were used, human (female) cervical cancer HeLa cells (ATCC) and human (female) osteosarcoma U2OS (ATCC). The cells were cultured at 95% humidity, 5% CO_2_ and 37°C in DMEM Glutamax high glucose (Life Technologies, Gibco, #31966-021) supplemented with 1% (v/v) penicillin-streptomycin (PEST) (Corning, #30-002-Cl) and 10% (v/v) fetal bovine serum (FBS) (Life Technologies, Gibco, #10500-064). All cell lines are screened for mycoplasma contamination on a regular basis.

### Stable cell lines

To make stable Samp1a–YFP cell lines, HeLa and U2OS cells were transfected with the pEYFP-N1-Samp1aYFP plasmid (Buch et al., 2009); stable clones were selected and maintained in culture medium supplemented with 200 µg/ml G418 and 10% (v/v) FBS.

### siRNA

At 24 h before transfection cells were seeded in tissue culture plates with glass coverslips. On the day of transfection, the cell culture medium was replaced with fresh medium (DMEM, 1% PEST, 10% FBS). siRNA oligonucleotides were incubated together with HiPerFect (1:1 nM/μl) transfection reagent (QIAGEN, #301705) in serum-free medium for 30 min at room temperature (18–20°C). Cells were treated with siRNA (18 nM) for 96 h. For siRNA-mediated post-transcriptional silencing of Samp1, we used dsRNA oligonucleotides (Ambion) corresponding to the following sense sequences: siSamp1 oligo1, 5′-GCGGCUGUGGAGUACUACAtt-3′, directed against exon 4 of the Samp1 mRNA targeting both the overexpressed Samp1a–YFP and all endogenous Samp1 isoforms; siSamp1a oligo2, 5′-GGAAGUGUUGACAGUGUGAtt-3′ and siSamp1a oligo3, 5′-UGCUUGCGUCUCGAAUCUUtt-3′ both directed against the 3′UTR of Samp1 mRNA and thus specific for the short splice variant (Samp1a) of endogenous Samp1. siSamp1a oligo2 and oligo3 do not target the recombinant protein Samp1a–YFP. As a control, we used scrambled Stealth RNAi™ siRNA negative control, medium GC content (Invitrogen, #12935300).

### Fluorescence activated cell sorting

siRNA-treated HeLa cells were grown as described above. Non-adhering cells were collected by centrifugation (800 ***g*** for 5 min). Adherent cells were washed in PBS and trypsinised at 37°C for 5 min. The trypsinised cells were diluted ten times in PBS, combined with the non-adherent cells and pelleted by centrifugation 800 ***g*** for 5 min. The pellets were suspended in 0.5 ml PBS and cells were fixed in 4.5 ml 70% ethanol at 4°C, overnight. The fixative was removed by centrifugation at 800 ***g*** for 5 min and the pellet was dissolved in PBS containing 20 µg/ml propidium iodide (PI), 200 µg DNase-free RNase and 0.1% Triton X-100, and incubated 15 min on ice. Before loading samples on to the FACS machine (FACScalibur, Becton Dickinson), the cells were re-suspended by pipetting. PI-stained DNA content was evaluated using a green diode 488 nm laser line for cell cycle analysis.

### Immunofluorescence

The following antibodies, buffers and fixation protocols were used for immunofluorescence. Primary antibodies were as follows: mouse anti-β-tubulin E7, 1:500 dilution (Developmental Studies Hybridoma Bank) MES buffer and methanol (MeOH) fixation; rabbit polyclonal anti-Samp1, 1:500 dilution (Buch et al., 2009), PBS or MES buffer and formaldehyde fixation; mouse anti-pericentrin, 1:1000 dilution (Abcam, #ab28144), PBS or MES buffer, formaldehyde or MeOH fixation; mouse anti-γ-tubulin, 1:500 dilution (Sigma-Aldrich, #T6557, clone GTU-88), MES buffer and formaldehyde fixation; and rabbit anti-HAUS6 (Augmin), 1:500 dilution (GeneTex, #GTX118732), PBS or MES buffer and MeOH fixation. Secondary antibodies were as follows: Alexa Fluor 488-conjugated donkey anti-mouse IgG, 1:5000 dilution (Invitrogen, #A21202); Alexa Fluor 568-conjugated donkey anti-rabbit IgG, 1:5000 dilution (Invitrogen, #A11011); Alexa Fluor 488-conjugated goat anti-rabbit IgG, 1:5000 dilution (Invitrogen, #A110008); and Alexa Fluor 568-conjugated goat anti-mouse IgG, 1:5000 dilution (Invitrogen, #A11004).

Buffers used were as follows: PBS buffer, 1× phosphate-buffered saline (PBS tablets; Life Technologies, Gibco, #18912-014); MES buffer, MES cytoskeleton buffer (10 mM MES, 138 mM KCl, 3 mM MgCl_2_, 2 mM EGTA, pH 7.2). Formaldehyde fixation was carried out by washing coverslips twice in buffer at 4°C, followed by fixation in 4% formaldehyde in buffer on ice for 20 min and permeabilisation with 0.5% Triton X-100 in buffer for 8 min on ice. MeOH fixation was carried out by washing coverslips twice in buffer at room temperature (18–20°C), followed by fixation and permeabilisation in −20°C MeOH at room temperature (18–20°C) for 5 min.

The general protocol for immunofluorescence was to grow cells on 13 mm, no. 1.5 glass-coverslips (VWR, #631-0150), which were then fixed and permeabilised according to the specific antibody protocol (see above). Thereafter, the cells were washed twice with buffer and blocked for 1 h in buffer containing 0.1% Tween-20 (buffer-T; PBS-T or MES-T as highlighted in the general protocol) and 2% BSA. Primary antibodies were diluted in buffer-T with 2% BSA and incubated for 1 h at room temperature (18–20°C) followed by four washes in buffer-T with 2% BSA. Secondary antibodies were diluted in buffer-T with 2% BSA, and 1 µM DRAQ5 (Biostatus, Thermo Fisher Scientific, #62251) DNA stain and incubated for 1 h at room temperature (18–20°C). The coverslips were then incubated and washed with buffer-T containing 5 µM Hoechst 33342 (Molecular Probes) at room temperature for 6 min. The coverslips were washed three times for 5 min each time at room temperature (18–20°C) in buffer-T before mounting on objective glass using Fluoromount G (SouthernBiotech). Nail polish was used to seal the coverslips.

### Image acquisition and processing

Acquisition of Samp1 and SiR–tubulin in live cells, and *z*-stacks for visualisation of γ-tubulin, Augmin (HAUS6) and Samp1 in the mitotic spindle (Figs 1, 2B and 4–6) was performed by using a Zeiss Axiovert 200 inverted microscope and a 100×1.3 NA oil-immersion objective, coupled to a Perkin Elmer spinning disk Yokugawa csu22 confocal microscope. For fluorophore excitation, argon/krypton laser lines 488 nm, 568 nm and 647 nm were used. For phase-contrast and fluorescence live-cell time-lapse imaging a Leica TCS-SP1 laser-scanning confocal microscope with a climate chamber (Ludin) was used with a 20× objective or a 63×1.4 NA oil immersion objective, respectively (Fig. 3). For scoring the nuclei morphology phenotypes (Fig. 2B,C), a Leica DMIRBE2 Epi-fluorescence microscope with a 40×1.25 NA oil-immersion objective was used. For automatic image acquisition of interphase cell nuclei (Fig. 2E,F), a Zeiss LSM 780 confocal microscope with a 20×0.8 NA Plan-Apo objective was used. For quantification of the fluorescence signal (Figs 4A,B and 6A–F) the image analysis software program ImageJ was used. A region of interest (ROI), for example, the perimeter of the metaphase plate was drawn. The average pixel values in the ROI was determined in the channel for β-tubulin, γ-tubulin and/or Augmin, by using the ‘Analyze-measure’ tool. The ROI mean intensity values were compared between the different treatments. All microscopy images have been processed in ImageJ; the contrast and brightness were set to the same values in the different treatments, any filter used is stated in the text for respective image.

### Live-cell imaging

For live-cell imaging (Figs 1 and 3), cells were grown in a dish with a glass bottom dish in DMEM/F12 containing HEPES (15 mM) (Life Technologies, Gibco, #11330-032), and supplemented with 10% (v/v) FBS and 1% (v/v) PEST. Microtubules (Fig. 1B–D) were probed for by using the cell permeable microtubule probe SiR–tubulin (tebu-bio, SPIROCHROME, #SC002). The cells were incubated for 24 h with SiR–tubulin (50 nM) in culture medium before being synchronised at the G2/M boundary by means of the CDK1 inhibitor RO-3306 (14 μM) (Sigma-Aldrich, #SML0569) diluted in cell culture medium for 16 h. The cells were washed and released from RO-3306 into fresh DMEM/F12 supplemented with 25 nM SiR–tubulin. Live-cell imaging started 2–3 h after the RO-3306 release. A climate chamber was used to keep the temperature at 37°C. The imaging sampling occurred for 1–2 h (Fig. 1). The cells in Fig. 3 were not synchronised, and image sampling occurred overnight. In Fig. 3B HeLa cells stably expressing GFP–tubulin and H2B–mCherry (Mackay et al., 2010) were used.

### Synchronisation of cells

For MCLIP, cells were first synchronised in S-phase by means of a 24 h thymidine block (2 mM) followed by a 7 h release into fresh cell culture medium. The cells were then synchronised into prometaphase by incubation in 100 ng/ml Nocodazole in DMSO (Sigma-Aldrich, #M1404) diluted in cell culture medium for 9 h. Prometaphase cells were collected by mitotic shake-off. The mitotic shake-off cells were washed free from nocodazole and replated in fresh medium containing 25 µM of the proteasome inhibitor MG-132­ in DMSO (Selleckchem.com, #S2619). The cells were grown until methaphase plates were visible by phase contrast microscopy (∼3 h later). The metaphase cells were collected for immunoprecipitation.

For immunofluorescence, cells were grown on coverslips and synchronised into the G2/M boundary by using the CDK1 inhibitor RO3306 (14 μM) (Sigma-Aldrich, #SML0569) diluted in cell culture medium for 16 h. The cells were released from RO3306 and incubated in fresh culture medium containing 25 µM MG132. The cells were grown until metaphase plates were visible by phase-contrast microscopy (∼3 h later). The synchronised cells were subjected to the immunofluorescence protocol, described above*.*

### Membrane protein cross-link immunoprecipitation

To detect protein–protein interactions of ‘hard-to-extract’ transmembrane proteins of the nuclear envelope we used our previously described MCLIP method (Jafferali et al., 2014). U2OS Samp1a–YFP-expressing cells were synchronised either in prometaphase or in metaphase. Asynchronous cultures were used to represent interphase cells. Cells were treated with the cell permeable reversible *in vivo* cross-linker, dithiobis-succinimidyl-propionate (DSP, Pierce/Thermo Fisher Scientific, #22585), at a final concentration of 1 mM in cell culture medium for 15 min at room temperature (18–20°C). After crosslinking, the cells were collected by centrifugation at 800 ***g*** for 10 min and quenched with 15 mM Tris-HCl pH 7.4 for 10 min at room temperature and washed twice with ice-cold PBS. The cell pellets were re-suspended in five volumes of 7 M urea and 1% Triton X-100 containing protease inhibitor cocktail (Calbiochem, #53914), incubated on ice for 20 min and homogenised with a 23-gauge needle syringe. A part of the lysate was saved as the input and combined with an equal volume of 2× sample buffer (100 mM Tris-HCl, 4% SDS, 20% glycerol, 0.04% Bromophenol Blue, 200 mM DTT) and boiled for 10 min. The remaining lysate was diluted 8× with PBS with protease inhibitor and sonicated on ice five times for 5 s each time and cleared by centrifugation at 800 ***g*** for 5 min. The sonicates were then pre-incubated with 2% BSA-blocked protein-G–Sepharose beads (control) for 1 h at 4°C with end-over-end rotation. The pre-incubated lysate was then added to agarose beads covalently coupled to camel GFP trap antibodies (Chromotech, #gta-20) for 2 h at 4°C with end-over-end rotation. The anti-GFP beads were washed twice with wash buffer (250 mM NaCl, 10 mM Hepes, 0.5% Triton X-100, pH 7.4), the bound proteins were released, and the crosslink was reversed by addition of equal volume of 2× sample buffer containing DTT followed by incubation for 10 min at 95°C. The unbound fraction from GFP trap was concentrated by trichloroacetic acid (TCA) precipitation.

### Western blot analysis

Cultured cells were suspended and pelleted by centrifugation at 800 ***g*** for 5 min. The pellets were dissolved in a small volume of PBS and an equal volume of 2× sample buffer (100 mM Tris-HCl, 4% SDS, 20% glycerol, 0.04% Bromophenol Blue and 200 mM DTT) was added. Cell lysates were boiled for 10 min at 95°C and stored at −70°C until use. Equal amounts were loaded on precast 10% SDS-PAGE acrylamide gels (Bio-Rad). SDS-PAGE-separated proteins were transferred onto MeOH-activated PVDF membranes, blocked with 5% milk in PBS with 0.1% Tween 20 (PBS-T), incubated with primary antibodies for 1 h, washed four times for 15 min with blocking buffer and then incubated with secondary antibodies for 1 h. After additional washing in PBS-T, PVDF membranes were subjected to ECL detection (Supersignal west dura, Thermo Fisher Scientific) and analysed using the ChemiDoc XRS+; imaging system (Bio-Rad). The following antibodies were used. Primary antibodies were: rabbit anti-Lamin-B1, 1:1000 (Abcam, #ab16048); mouse anti-β-tubulin E7, 1:500 (Developmental Studies Hybridoma Bank); Rabbit polyclonal anti-Samp1, 1:500 (Buch et al., 2009); mouse anti-γ-tubulin, 1:500 (Sigma-Aldrich, #T6557, clone GTU-88); and rabbit anti-HAUS6 (Augmin), 1:500 (GeneTex, #GTX118732). Secondary antibodies were: horseradish peroxidase (HRP)-conjugated anti-rabbit-IgG (GE Healthcare, #NA934V), 1:5000; and HRP-conjugated anti-mouse-IgG (GE Healthcare, #NXA931V), 1:5000.

### Pulldown experiments

Bacterial cells (5×10^9^) expressing Strep-*Ct*.Samp1(1-180) (Vijayaraghavan et al., 2016) were treated with 1 ml of lysis buffer (100 mM Hepes, 200 mM NaCl, 1 mg/ml lysozyme, 10% glycerol and protease inhibitor, pH 7.4) on ice for 45 min. The bacterial cells were sheared by brief sonication and soluble proteins were recovered in the supernatant following centrifugation at 15,500 ***g*** for 30 min at 4°C. 50 μl of settled strep-tactin agarose beads (www.iba-lifesciences.com, #2-1201-002) were blocked with 5% BSA in PBS. To the blocked beads, 25 μmol of bait protein [Strep-*Ct*.Samp1(1-180); Vijayaraghavan et al., 2016] was added and the total volume was brought to 500 μl by addition of lysis buffer and incubated at 4°C for 1 h with end-over-end rotation. The unbound bacterial cell lysate was separated by centrifugation at 800 ***g*** for 3 min. To the beads, 25 μmol of prey protein (His-tag-γ-tubulin, Abcam, #ab115710) was added along with lysis buffer as before and incubated at 4°C for 1 h with end-over-end rotation. The beads were washed three times with wash buffer (100 mM Hepes, 250 mM NaCl, 0.5% Triton X-100, pH 7.4). The unbound lysates containing both bait and prey proteins were TCA precipitated. Equivalent amounts of bound, unbound and lysate fractions were loaded onto 10% SDS-PAGE precast gels (Bio-Rad, #456–1094) and analysed by western blotting using mouse monoclonal anti-His (1:2500) antibody (Thermo Fisher Scientific, #MA121315).

### Quantification and statistics analysis

All statistical analysis was performed using Microsoft Excel software and unpaired two-tailed Student's *t*-test. All replicates are biological replicates. The bar graphs show the mean±s.d. from three biological replicates. In the box plots, the whiskers shows the minimum and maximum values of the distributions, respectively. The lower and upper boxes represent the 25th and 75th percentiles, respectively, and the horizontal bar inside the boxes indicate the median. The box plots show a representative graph from one of the different biological replicates.

## Supplementary Material

Supplementary information
