# Supplementary material for: Mitotic spindle assembly and γ-tubulin localisation depend on the integral nuclear membrane protein Samp1
Source: J Cell Sci. 2018 Apr 13;131(8):jcs211664. doi: 10.1242/jcs.211664 (PMC5963844; doi:10.1242/jcs.211664)
Supplement: Supplementary information [file joces-131-211664-s1.pdf]

SUPPLEMENTARY FIGURES

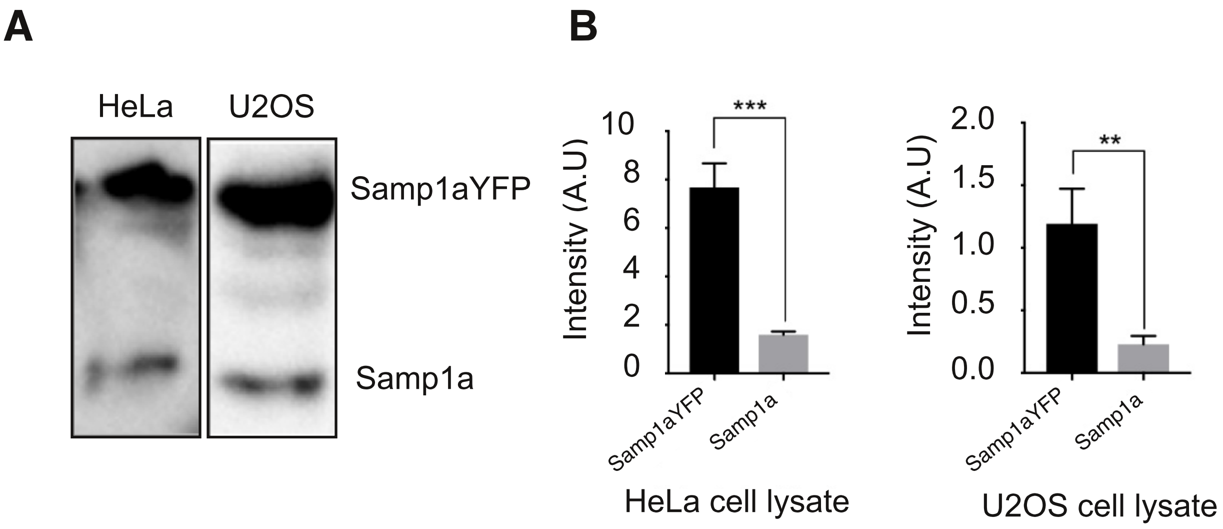

Figure S1. A) Endogenous Samp1a and recombinant Samp1aYFP protein levels in HeLa and U2OS cell lines. B) Quantification of Samp1a and Samp1aYFP expression levels in HeLa and U2OS cell lines (n=3 blots, mean ± S.D., unpaired two-tailed Student´s t-test).

Journal of Cell Science • Supplementary information

Journal of Cell Science • Supplementary information

SUPPLEMENTARY MOVIES

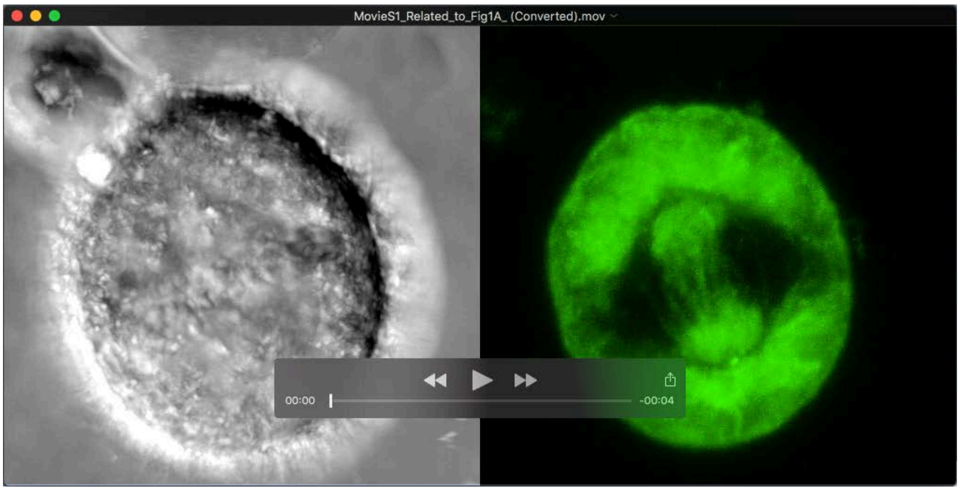

Movie 1. Related to figure 1B. Time-lapse movie showing a mitotic HeLa cell stably expressing Samp1aYFP, enhanced to visualize GFP fluorescence in the mitotic spindle. Frame rate 3 min. Scale bar 10μm.

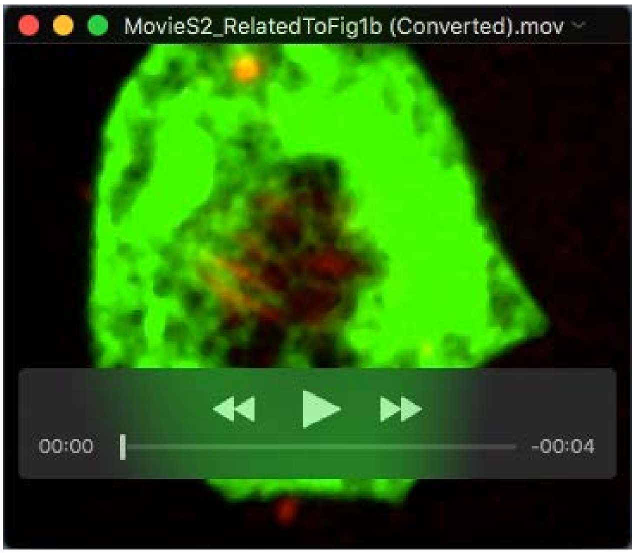

Movie 2. Related to figure 1C. Time-lapse movie showing a mitotic U2OS cell stably expressing Samp1aYFP and probed with SiR-tubulin to visualize microtubules.

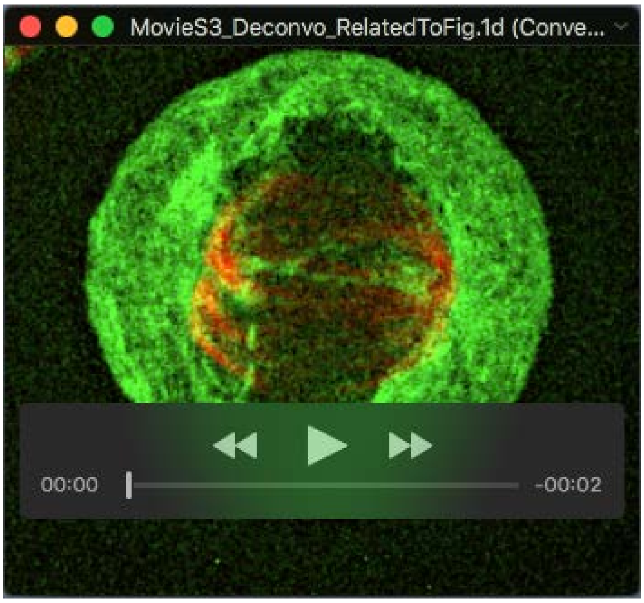

Movie 3. Related to figure 1E. Time-lapse movie showing a mitotic HeLa cell stably expressing Samp1aYFP and probed with SiR-tubulin to visualize microtubules. De-convolution was used to enhance the structures of Samp1aYFP and microtubules of the mitotic spindle.

Journal of Cell Science • Supplementary information
